# The Ethics of Medical Visiting

**Published:** 1889-01

**Authors:** Lawson Tait

**Affiliations:** Birmingham, Eng.


### Correspondence.

### *THE ETHICS OF MEDICAL VISITING.*

*To the Editor Buffalo Medical and Surgical Journal:*

SIR—I must, first of all, express my deep obligation to your editorial fairness in the remarks which you make in the December number of your JOURNAL, upon the correspondence which has occurred between myself and Dr. Steele, of Chicago. Up to the present time, there has existed an unwritten code, by which members of the medical profession visiting in another country, or another district, have always been welcomed at the clientéles of practitioners engaged in specially prominent positions. The time, however, is clearly arising when some new kind of understanding has to be arrived at upon this point, and it is really with very great regret indeed that I have to indicate for myself a departure from what may be, at least, considered an established custom. I can very well remember the first time when an American visitor applied to me for access to my work, and when I say it was no less a distinguished person than Dr. Levis, of Philadelphia, some eighteen years ago, I can be excused for saying that I felt the request to be a compliment. From that time till some two years ago, visitors from your continent came to me in large numbers, and were always welcomed not only as spectators in my operating-room and in my wards but to my table and to my house. This went so far, that my house was nicknamed by my neighbors "The Stars and Stripes Hotel." There was hardly a week—during a certain part of the year there was not a day—passed, in which some American visitor or visitors were not entertained; and I have not had cause to regret my reception of them, except in a single instance. The exercise of hospitality became so general, and so misunderstood, that many of these casual visitors, without any kind of introduction, claimed it—claimed everything I had to give them—as a matter of right, and expressed themselves absolutely injured if I did not alter the whole of my arrangements to meet their exigencies of travel.

Another matter upon which errors of a most extraordinary kind, from our English point of view, were made, was that a gentleman from America arrived here armed with a letter of introduction from another American practitioner, quite as unknown to me as was he who presented the credential.

In this part of the world we understand that a letter of introduction is sent to introduce an unknown person by one who is quite well known to the receiver of the letter, but in these cases the recommender and recommended were equally unknown. But at this time a change came over the spirit of affairs, and I found that my hospitable intentions were being abused; that what I had done for the purpose of advancing the art I practise, was really becoming a mischief. In the first place, visitors became far too numerous for me to undertake the duty of receiving them. They crowded my small wards, they hampered my operative proceedings, and they came to be of a quality which clearly indicated that I was doing more harm than good. This was forcibly impressed upon me by reminders which I received by word of mouth and by written communications from some of the most eminent practitioners in America, that I was encouraging incompetent and ill-educated men to engage in work for which they were qualified neither by training nor experience. They came to see me, they saw a difficult operation completed in a few minutes, and they went away with the belief that without the amount of troubles which I had myself experienced, they might by some unknown royal road achieve the same success. The result was the so-called "laparatomy epidemic" which spread all over your continent, and of which your best writers now bitterly complain. On the other hand, a number of serious students came to settle with me for longer periods, and then went away with a just title to have understood, appreciated, and to be able to follow my work. Of these, I am pleased to say, I can now number some twelve or fifteen, whose brilliant results entirely justify the words I use concerning them. But even to these men I was doing an injustice, for I found that if Dr. Smith, of Smithville, had been my pupil for six months, his rival, Dr. Jones, of the same city, having been here for six hours, talked as loudly, in fact more loudly, of his knowledge of my methods and reasons of my success than he who had been with me a diligent student for a long period. Finally, there arrived a class of men who were not only ignorant, but conceited of that confidence begotten of ignorance, not only assumed that they knew everything, but misrepresented what they saw. Of these, you have given some admirable examples. I cannot imagine anything more objectionable, nor anything more untrue; than the statement which Dr. Steele has made of Mr. Treves, Mr. Christopher Heath and Mr. Knowsley Thornton; and their objectionable nature is not diminished when we find that they are made chiefly for the purpose of eliciting an approval from Dr. Steele, of his own unexampled powers.

A notable instance of this forced itself upon me in the shape of Dr. Senn, of Milwaukee, who, as I have already said, was here in my house for a few hours, and managed to completely misunderstand everything he saw and everything I told him, and to misrepresent me in a most complete and unsatisfactory fashion. Another visitor, whose name I shall not indicate, because, so far, it has not become public, did even worse than this—he committed an offence which, on this side of the Atlantic, is considered a sin of the deepest dye; he filched from me by pecuniary bribes one of my best and most trusted servants with the hope, which I trust has not been unfulfilled, that she would serve him to achieve the desired success. This event has not been single; it has been repeated against myself and against others. Another and a different kind of offence consists that in having introduced these visitors to my medical and surgical colleagues in this town and neighborhood, they have indulged in that habit of interviewing, so common on your side, but which we regard as very annoying; and in characteristic contributions which have resulted from these interviews, they have indulged in comments on the personal appearance and habits of professional practitioners of this town, of a kind which, to Englishmen, is extremely offensive. To you such comments are, I know, matters of every-day occurrence. Here they are comparatively unknown, and when they are experienced they are always bitterly resented.

For such reasons as I have given, it need not be a matter of surprise that, for the last two years, I have come to the determination to exclude all casual visitors from my practice, and it is a determination which has been arrived at by others as well as myself. We cannot imagine how any good can be done from witnessing an occasional operation, and when visitors evince the spirit which has characterized Dr. Senn and Dr. Steele, I must, on behalf of my brethren on this side, confirm the opinion which you so freely express, that the controversy will influence British and continental surgeons in a most unfortunate direction.

LAWSON TAIT.

BIRMINGHAM, ENG., Dec. 15, 1888.

